# A Composite Biomarker of Derived Neutrophil–Lymphocyte Ratio and Platelet–Lymphocyte Ratio Correlates With Outcomes in Advanced Gastric Cancer Patients Treated With Anti-PD-1 Antibodies

**DOI:** 10.3389/fonc.2021.798415

**Published:** 2022-02-18

**Authors:** Yuting Pan, Haiyan Si, Guochao Deng, Shiyun Chen, Nan Zhang, Qian Zhou, ZhiKuan Wang, Guanghai Dai

**Affiliations:** ^1^ Chinese People’s Liberation Army Medical School, Beijing, China; ^2^ Medical Oncology Department, The First Medical Center, Chinese People’s Liberation Army General Hospital, Beijing, China

**Keywords:** immunotherapy, advanced gastric cancer, dNLR, PLR, efficacy, prognosis

## Abstract

**Background:**

The highly heterogeneous characteristics of GC may limit the accuracy of a single biomarker for screening populations benefiting from immunotherapy. However, the combination of multiple indicators can provide more directed information for the detection of potential immune benefit subgroups. At present, there are no recognized complex indexes to identify advanced GC (AGC) in patients who likely benefited from immunotherapy. The objective of this research is to explore whether the composite biomarker of derived neutrophil–lymphocyte ratio (dNLR) and platelet–lymphocyte ratio (PLR) can be used as a reliable prognostic factor for the survival of AGC patients receiving immunotherapy.

**Methods:**

From December 2014 to May 2021, a total 238 AGC patients at a single Center were included in this retrospective cohort research study. The cutoff value of dNLR was obtained by the ROC curves to predict the disease progression rate at the 8th month and the cutoff value of PLR was estimated by the median value. The cutoff values of dNLR and PLR were 1.95 and 163.63, respectively. The high levels of dNLR (≥1.95) and PLR (≥163.63) were considered to be risk factors. Based on these two risk factors, patients were categorized into 3 groups: the risk factor number for the “good” group was 0, that for the “intermediate” group was 1, and that for the “poor” group was 2. The subjects were divided into two groups: dNLR/PLR-good and dNLR/PLR-intermediate/poor.

**Results:**

Of the 238 patients, the median overall survival (mOS) and progression-free survival (mPFS) were 12.5 and 4.7 months, respectively. Multivariate analysis revealed that the good dNLR/PLR group was independently associated with better prognosis. The intermediate/poor dNLR/PLR group was independently correlated with an over 1.4 times greater risk of disease progression (4.1 months vs. 5.5 months; *p* = 0.016) and an over 1.54 times greater risk of death (11.1 months vs. 26.3 months; *p* = 0.033) than the good dNLR/PLR group. However, no clear differences in the disease control rate (DCR) and overall response rate (ORR) were observed between the intermediate/poor dNLR/PLR group and the good dNLR/PLR group (51.5% vs. 56.3%, 26.3% vs. 29.6%; *p* = 0.494, *p* = 0.609).

**Conclusion:**

Our study firstly verifies that the composite biomarker of dNLR and PLR is an independent prognostic factor affecting survival of advanced AGC patients receiving immunotherapy. It may be difficult for patients with the intermediate/poor dNLR/PLR group to benefit from immunotherapy.

## Introduction

Gastric cancer (GC) is one type of very common gastrointestinal tumors around the world. The incidence rate of GC ranks fifth globally and second domestically in China (1). The progress of GC treatment is relatively slow, and traditional chemotherapy, such as surgery, chemotherapy, and targeted therapy, is therefore limited. The emergence of immunotherapy brings a new option for GC; nevertheless, its application in GC is still difficult. Researchers have been trying both back line and front line, as well as single-drug and different combinations. The “ATTRACTION-2” study confirms the efficacy of nivolumab for the back line of GC (2, 3). The results of the “KEYNOTE-061” study were negative, and pembrolizumab failed in the second-line chemotherapy challenge (4–6). The “CheckMate 649” study explored whether the nivolumab-based first-line immunotherapy was suitable for advanced GC (AGC) (7). Moehler et al. found that patients treated with a combination of nivolumab and chemotherapy showed consistent overall survival (OS) benefits in the whole population and the Chinese subgroup, regardless of the expression status of programmed death ligand-1 (*PD-L1*) (8). The first result of the “KEYNOTE-811” study showed that *HER2*-positive metastatic gastric or gastroesophageal junction cancer could benefit from using the combination of pembrolizumab, trastuzumab, and chemotherapy (9). However, the current evaluation of biomarkers for immunotherapy has been limited. There is a lack of effective biomarkers that can be used as prognostic factors for AGC-treated patients with immune checkpoint inhibitors (ICIs). In recent years, the expression of *PD-L1* and microsatellite steady-state (MSI) in AGC patients can be effectively assessed for the efficacy of immunotherapy (10, 11).

Peripheral inflammatory blood complex index such as neutrophil–lymphocyte ratio (NLR), platelet–lymphocyte ratio (PLR), and hemoglobin (Hb) levels have demonstrated a promising and suitable biomarker prognostic for GC (12–15). However, the highly heterogeneous characteristics of GC may limit the accuracy of a single biomarker for screening populations benefiting from immunotherapy. In contrast, the combination of multiple indicators can provide more targeted information for the detection of potential immune benefit subgroups. Dharmapuri S et al. established a statistical model by NLR/PLR groups and found that the high-NLR/ low-PLR group in advanced hepatocellular carcinoma patients treated with anti–PD-1 therapy has shorter OS and progression free survival (PFS) than the low-NLR/ low-PLR group (16). However, as a biomarker of poor prognosis in AGC patients undergoing immunotherapy, the role of NLR is quite well defined in some studies (17–19). Furthermore, in May 2021, a study conducted by Lim et al. showed that non-small-cell lung cancer (NSCLC) patients with a high level of derived neutrophil–lymphocyte ratio (dNLR) baseline value were associated with poor outcomes, when they were treated with ICIs (20).

Our research found that patients with an elevated dNLR value (≥ the best cutoff value) were associated with shorter OS and PFS too. However, patients with high levels of PLR (≥ the median value) were only associated with shorter OS, but not PFS. Thus, we combined dNLR and PLR to stratify risk factors. The high levels of dNLR (≥1.95) and PLR (≥163.63) were considered to be risk factors. Based on these two risk factors, patients were categorized into 3 groups: the risk factor number for the “good” group was 0, that for the “intermediate” group was 1, and that for the “poor” group was 2. The subjects were divided into two groups: dNLR/PLR-good and dNLR/PLR-intermediate/poor. We then began to evaluate the differences in prognosis and survival of AGC patients after immunotherapy between the good and the intermediate/poor groups.

## Materials and Methods

### Study Population

From December 2014 to May 2021, all patients involved were diagnosed with GC at stage IV and received ICI treatment in the Senior Department of Oncology at Chinese PLA General Hospital. We set the inclusion criteria as follows: (1) patients detected with measurable lesions, (2) patients who underwent blood routine and blood biochemistry tests within 1 week before ICI administration, and (3) patients who have received at least two rounds of ICI treatment continuously. Patients failing to provide imaging data for comparing the efficacy of ICIs before and after treatment were excluded. As a result, a total of 238 patients were considered eligible for this cohort study. Clinical parameters of those AGC patients from their medical records were collected, including sex, age, Eastern Cooperative Oncology Group performance status scores (ECOG PS), smoking history, smoking exposure, history of diabetes, tumor type, the status of *HER-2* expression, the dose of ICIs, the status of bone metastasis, the status of liver metastasis, response to line before immunotherapy, the status of pleural fluid, the status of ascites, the number of metastatic sites, lines of treatment with ICIs, ICIs agent, and immunotherapy scheme. Meanwhile, blood parameters were analyzed routinely, including absolute neutrophil count (ANC), absolute lymphocyte count (ALC), white blood count cells (WBC), and platelet count (PLT) 7 days before immunotherapy implementing to obtain dNLR and PLR values.

### Treatment Regimens

Five treatment methods were used in this research study: (1) ICI monotherapy, (2) ICIs combined with chemotherapy, (3) ICIs combined with anti-angiogenic therapy, (4) ICIs combined with DNA-derived humanized monoclonal antibodies (trastuzumab) and chemotherapy, and (5) ICIs combined with chemotherapy and anti-angiogenic therapy. The types and doses of ICIs were as follows: (1) Sintilimab was injected intravenously 200 mg once every 3 weeks. (2) Toripalimab was injected intravenously 240 mg once every 3 weeks. (3) The recommended dose of pembrolizumab injection for intravenous infusion was a dose of 3 mg/kg, administered once every 3 weeks. (4) The recommended dose of nivolumab injection for intravenous infusion was a dose of 2 mg/kg, administered once every 2 weeks. The first imaging evaluation of nivolumab was carried out 2–4 weeks after the 3rd intravenous injection; nevertheless, the evaluation of toripalimab, sintilimab, and pembrolizumab was carried out 3–5 weeks after the 2nd intravenous injection. The trastuzumab course was administered for 3 weeks. For the first course, the dose was 8 mg/kg, applied by intravenous injection for 90 min. Starting from the 2nd course, the dose was lowered to 6 mg/kg. For the infusion time, if the patients tolerate trastuzumab well in the first course, the 2nd course was applied by intravenous injection for 30 min. course. Anti-angiogenic drugs involved were apatinib (850 mg, orally administrated 30 min after a meal, once a day) and bevacizumab (5 mg/kg body weight, once every 2 weeks; or 7.5 mg/kg body weight, once every 3 weeks). The chemotherapy regimens include (1) XELOX regimen: capecitabine (1,000 mg/m²) was used 2 times a day orally after breakfast and dinner for 14 consecutive days with 7 days of rest as a treatment cycle. Oxaliplatin (130 mg/m²) was added on the first day of each cycle by intravenous injection. (2) SOX regimen: tiggio (40–60 mg) was used 2 times a day orally after breakfast and dinner for 14 consecutive days with 7 days of rest as a treatment cycle. Oxaliplatin (130 mg/m²) was added on the first day of each cycle by intravenous injection. (3) DCF regimen: docetaxel (75 mg/m²), cisplatin (75 mg/m²), and fluorouracil (750 mg/m²) were applied by intravenous injection. On the first day of every course, each course lasted 21 days. (4) The combined regimen of irinotecan and oxaliplatin: irinotecan (180 mg/m²) and oxaliplatin (130 mg/m²) were applied by intravenous injection. On the first day of every course, each course lasted 14 days. (5) The combined regimen of irinotecan and raltitrexed: irinotecan (180 mg/m²) and raltitrexed (3 mg/m²) were applied by intravenous injection. On the first day of every course, each course lasted 14 days. (6) Others. The choice of the above regimens was based on the patient’s pathological stage and general health conditions.

### Assessment

For effectiveness evaluation, the disease control rate (DCR) and the overall response rate (ORR) is termed as the percentage of patients with complete response (CR), partial response (PR), and stable disease (SD) and the percentage of patients with CR and PR, respectively. For prognosis analysis, OS and PFS are counted from the time of the immunotherapy beginning to death and the time between the onset of ICIs and the progression or death of the tumor, respectively.

### dNLR, PLR, and Statistical Models by dNLR/PLR Groups

**Figure 1:**
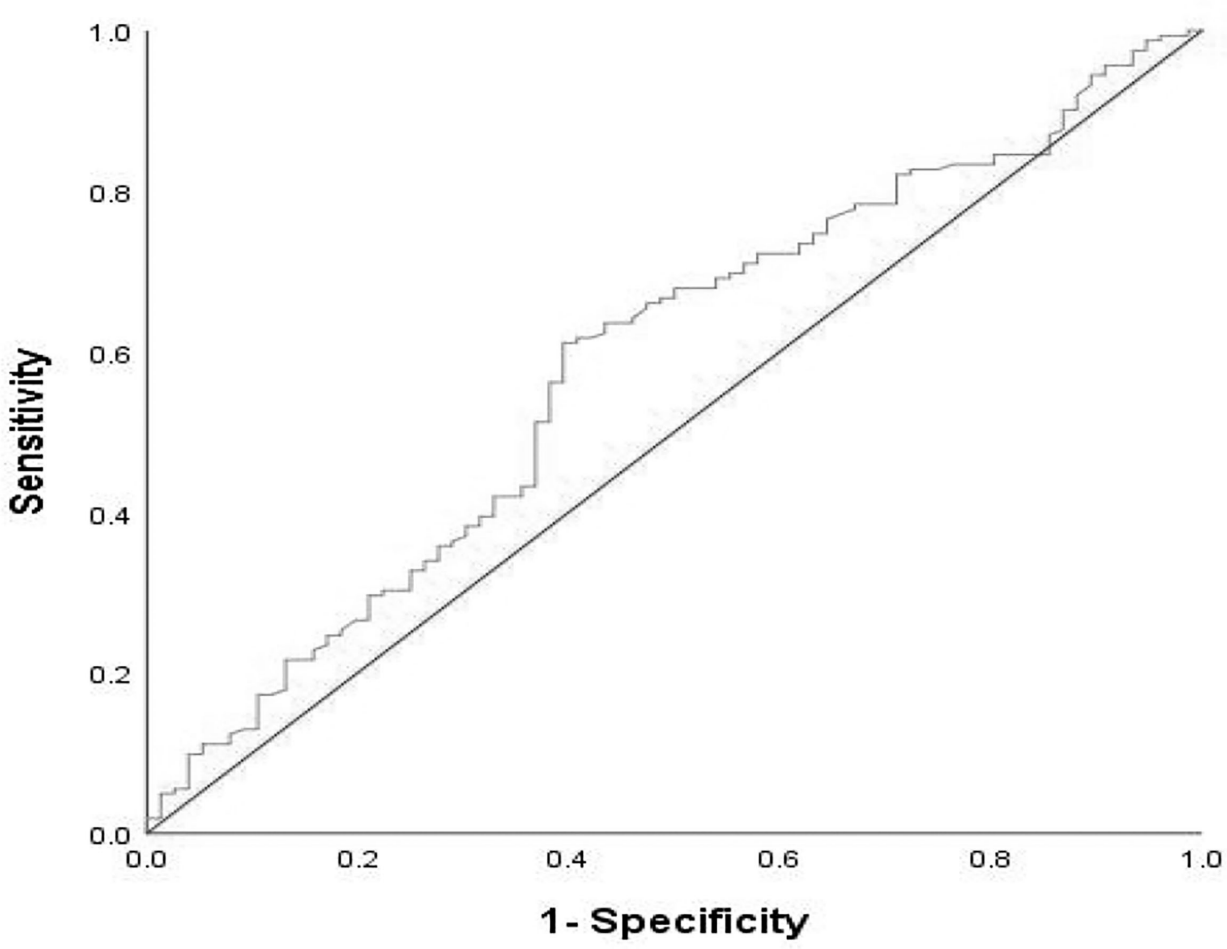
ROC curve of pretreatment dNLR in assessment of the disease progression rate at the 8th month. Sensitivity:61.1%; Specificity:60.5%; AUC:0.584; *p* = 0.037. dNLR, derived neutrophil–lymphocyte ratio; ROC, receive operating characteristic; AUC, areaunder the curve.

We analyzed the value of PLR (platelet/lymphocyte ratio) and NLR (neutrophil/lymphocyte ratio) 7 days before immunotherapy was implemented. With dNLR before treatment as the test variable, and the disease progression rate at the 8th month as the state variable, the receiver operating characteristic (ROC) curve of immunotherapy effect and dNLR level before treatment was drawn. The area under the ROC curve was 0.584, which indicated a statistically significant difference (*p* = 0.037). The best cutoff value of dNLR was 1.95, and its corresponding sensitivity and specificity were 61.1% and 60.5%, respectively (**Figure 1**). The cutoff value of PLR was estimated by the median value. The high levels of dNLR (≥ the best cutoff value) and PLR (≥ the median value) were considered to be risk factors. Based on these two risk factors, patients were categorized into 3 groups: the risk factor number for the “good” group was 0, and that for the “poor” group was 1 or 2. The risk factor number for the “good” group was 0, that for the “intermediate” group was 1, and that for the “poor” group was 2. Due to the similar efficacy and survival outcomes of patients in the intermediate and good groups (**Supplementary Table 1** and **Supplementary Figure 1**), we integrated the intermediate group into the poor group, forming the intermediate/poor group.

### Statistical Analysis

SPSS 26.0 software was used to perform all statistical analyses. Data were summarized as the median values for non-normally distributed continuous variables. Based on the values of *α* (*α* = 0.05) and *β* (*β* = 0.2), the expected median OS (mOS) of the good group and the intermediate/poor group, we evaluated the number of the sample size of our retrospective cohort study. the expected mOS of the good group and the intermediate/poor group were 17 and 11 months, respectively. Data were reported as percentages and counts for categorical variables. The ROC curves were applied to clarify the best cutoff value of dNLR. *χ* ^2^ or Fisher’s exact test was carried out to evaluate the relationship between clinical response and dNLR/PLR groups of AGC patients. The survival curve was depicted by Kaplan–Meier analysis. Logistic regression models and Cox proportional hazard were applied to assess the prognostic values of dNLR/PLR groups for DCR and survival, respectively. *p*-values less than 0.05 (*p* < 0.05) were considered statistically significant.

## Results

### Baseline Characteristics

A total of 238 AGC patients receiving ICIs were reviewed in the retrospective cohort study. The clinical features of patients are provided below (**Table 1**). The median age was 58 years. Among these patients, 121 patients were elders (≥58 years); 188 patients were male, 63 patients had Cardia, 99 had body/fundus, and 76 patients had pylorus cancer; 223 patients had ECOG PS scores of 0–1; 33 patients had positive *HER-2* expression, 163 patients had a negative expression, and 42 patients were untested; 118 patients had poor tumor differentiation, 101 patients had moderate tumor differentiation, 4 patients had good tumor differentiation, and tumor differentiation was unknown for 15 patients; 12 patients had pleural fluid; 54 patients had ascites; 22 patients had bone metastases before immunotherapy. After grouping, 71 patients were in the good dNLR/PLR group and 167 patients were in the intermediate/poor dNLR/PLR group.

**Table 1:** General data and clinical features.

Characteristics	Number of Patients (%)		
	Overall (*n* = 238)	The good group (*n* = 71)	The intermediate/poor group (*n* = 167)
Median age (range), years	58 (18–86)	58 (27–82)	58 (18–86)
Sex			
Female	62 (26.1)	21 (29.6)	41 (24.6)
Male	176 (73.9)	50 (70.4)	126 (75.4)
Smoking history			
Yes	88 (37)	26 (36.6)	62 (37.1)
No	150 (63)	45 (63.4)	105 (62.9)
Smoking exposure			
>30 packs per year	44 (18.5)	14 (19.7)	30 (18.0)
≤30 packs per year	194 (81.5)	57 (80.3)	137 (82.0)
Drinking history			
Yes	85 (35.7)	26 (36.6)	59 (35.3)
No	153 (64.3)	45 (63.4)	108 (64.7)
Tumor location			
Cardia	60 (25.2)	18 (25.4)	42 (25.1)
Body/Fundus	89 (37.4)	23 (32.4)	66 (39.5)
Pylorus	86 (36.1)	30 (42.3)	56 (33.5)
Unknown	3 (1.3)	0 (0)	3 (1.8)
Response to line before immunotherapy			
PD	159 (66.8)	40 (56.3)	119 (71.3)
Others	79 (33.2)	31 (43.7)	48 (28.7)
Liver metastasis			
Present	100 (42)	30 (42.3)	70 (41.9)
Absent	138 (58)	41 (57.7)	97 (58.1)
Pleural fluid			
Present	12 (5.0)	3 (4.2)	9 (5.4)
Absent	226(95)	68 (95.8)	158 (94.6)
Ascites			
Present	54 (22.7)	8 (11.3)	46 (27.5)
Absent	184 (77.3)	63 (88.7)	121 (72.5)
Bone metastasis			
Present	22 (9.2)	8 (11.3)	14(8.4)
Absent	216 (90.8)	63 (88.7)	153(91.6)
Number of metastatic sites			
≥3	58 (24.4)	11 (15.5)	47 (28.1)
<3	180 (75.6)	60 (84.5)	120 (71.9)
Dosage of immunotherapy			
≥200 mg	147 (61.8)	49 (69.0)	98 (58.7)
<200 mg	91 (38.2)	22 (31.0)	69 (41.3)
Tumor differentiation			
Poorly	176 (73.9)	50 (70.4)	126 (75.4)
Moderately	42 (17.6)	14 (19.7)	28 (16.8)
Well	4 (1.7)	3 (4.2)	1 (0.6)
Unknown	16 (6.7)	4 (5.6)	12 (7.2)
Lines of immunotherapy			
≥2	130 (54.6)	33 (46.5)	97 (58.1)
<2	108 (45.4)	38 (53.5)	70 (41.9)
ECOG PS			
≥2	15 (6.3)	1 (1.4)	14 (8.4)
0-1	223 (93.7)	70 (98.6)	153 (91.6)
PD-1 inhibition agent			
Nivolumab	84 (35.3)	25 (35.2)	59 (35.3)
Pembrolizumab	30 (12.6)	6 (8.5)	24 (14.4)
Others	124 (52.1)	40 (56.3)	84 (50.3)
Therapies			
ICIs monotherapy	52 (21.8%)	14 (19.7)	38 (22.8)
ICIs combined with other therapies	186 (78.2%)	57 (80.3)	129 (18.6)

PD, progressive disease; PD-1, programmed cell death-1; ECOG PS, Eastern Cooperative Oncology Group performance status scores; ICIs, immune checkpoint inhibitors.

### Treatment Characteristics

Of 238 patients, 158 (66.8%) patients had previously progressed before using ICIs; 84 (35.3%) patients received nivolumab, 30 (12.6%) patients were treated with pembrolizumab, and 124 (52.1%) patients received other immunotherapy drugs; 130 (54.6%) patients used the 1st line ICIs and 108 (45.4%) patients used ICIs after the 1st line; 186 (78.2%) patients were treated with ICIs combined with other therapies; 52 (21.8%) patients were treated with ICI monotherapy (**Table 1**).

### A Composite Biomarker of dNLR and PLR for Response to ICIs

The optimal efficacy of all AGC patients was evaluated in the study, and the results were as follows: 112 (47.1%) patients had progressive disease (PD), 4 (1.7%) patients had CR, 62 (26.1%) patients had PR, and 60 (25.2%) patients had SD. The ORR was 27.7% and DCR was 52.9% (**Table 2**). No clear differences in DCR and ORR were observed between the intermediate/poor dNLR/PLR group and the good dNLR/PLR group (51.5% vs. 56.3%, 26.3% vs. 29.6%; *p* = 0.494, *p* = 0.609) (**Table 2**).

**Table 2:** Relationship between the good group and the intermediate/poor group and response to ICIs treatment.

Best Overall Response	Number of Patients (%)			*p*-value
	Overall, *n* = 238	The good group, *n* =71	The intermediate/poor group, *n* = 167	
CR	4 (1.7)	2 (2.8)	2 (1.2)	0.388
PR	62 (26.1)	20 (28.2)	42 (25.1)	0.627
SD	60 (25.2)	18 (25.4)	42 (25.1)	0.974
PD	112 (47.1)	31 (43.7)	81 (48.5)	0.494
ORR	65 (27.3)	21 (29.6)	44 (26.3)	0.609
DCR	126 (52.9)	40 (56.3)	86 (51.5)	0.494

ICIs, immune checkpoint inhibitors; CR, complete response; PR, partial response; SD, stable disease; PD, progressive disease; DCR, disease control rate; ORR, overall response rate.

### dNLR and PLR for Survival of AGC Patients

**Figure 2:**
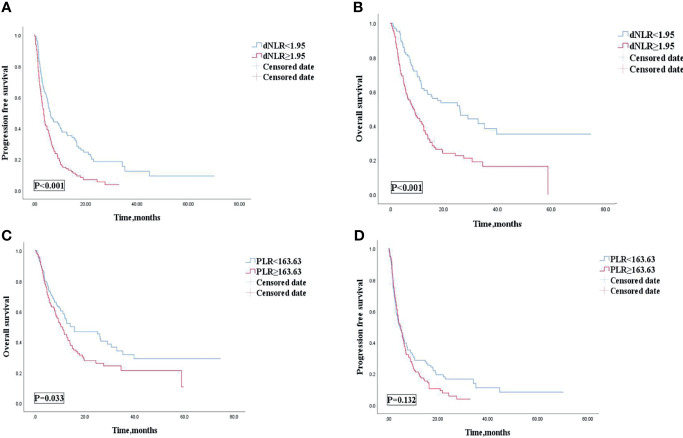
PFS **(A)** and OS **(B)** of the dNLR of patients with AGC receiving ICIs cohort, and OS **(C)** and PFS **(D)** of the PLR of patients with AGC, receiving ICIs cohort. PFS, progression free survival; OS, overall survival; AGC, advanced gastric cancer; ICIs, immune checkpoint inhibitors; dNLR, derived neutrophil‐to‐lymphocyte; PLR, platelet–lymphocyte ratio.

The cutoff value of dNLR and PLR were 1.95 and 163.63, respectively. For patients with an elevated dNLR value (≥1.95) and with a lower dNLR value (<1.95), the mPFS was 3.6 (95% CI, 2.855–4.345) and 6.2 (95% CI, 4.488–7.912) months, respectively, and the mOS was 9 (95% CI, 6.032–11.968) and 26 (95% CI, 14.286–37.714) months, respectively. Patients with an elevated dNLR value were associated with an over 1.8 times greater risk of disease progression (HR = 1.807; 95% CI, 1.356–2.407; *p* < 0.001) and an over 2.1 times greater risk of death (HR = 2.161; 95% CI, 1.542–3.028; *p* < 0.001) than those with a lower dNLR value (**Table 3** and **Figures 2A, B**). For patients with an elevated PLR value (≥163.63) and with a lower PLR value (<163.63), the median PFS was 4.6 (95% CI, 3.549–6.251) and 4.9 (95% CI, 2.983–6.017) months, respectively, and the mOS was 10.4 (95% CI, 7.386–13.414) and 15.8 (95% CI, 4.400–27.200) months, respectively. Patients with an elevated PLR value were associated with an over 1.4 times greater risk of death than those with a lower PLR value (<163.63) (HR = 1.416; 95% CI, 1.026–1.956; *p* = 0.033). However, no clear difference of PFS was observed between the two groups of patients (HR = 1.237; 95% CI, 0.936–1.636; *p* = 0.132) (**Table 3**; **Figures 2C, D**).

**Table 3:** Survival of dNLR and PLR.

Classification	PFS (months)			OS (months)		
	Median (95% CI)	HR (95% CI)	*p*	Median (95% CI)	HR (95% CI)	*p*
dNLR<1.95	6.2 (4.488–7.912)	1 [the reference]		26.0 (14.286–37.714)	1 [the reference]	
dNLR≥1.95	3.6 (2.855–4.345)	1.807 (1.356–2.407)	<0.001	9.0 (6.032–11.968)	2.161 (1.542–3.028)	<0.001
PLR<163.63	4.9 (2.983–6.017)	1 [the reference]		15.8 (4.400–27.200)	1 [the reference]	
PLR≥163.63	4.6 (3.549–6.251)	1.237 (0.936–1.636)	0.132	10.4 (7.386–13.414)	1.416 (1.026–1.956)	0.033

PFS, progression-free survival; OS, overall survival; HR, hazard ratio; dNLR, derived neutrophil‐to‐lymphocyte, PLR, platelet–lymphocyte ratio.

### The Composite Biomarker of dNLR and PLR for PFS of AGC Patients

**Figure 3:**
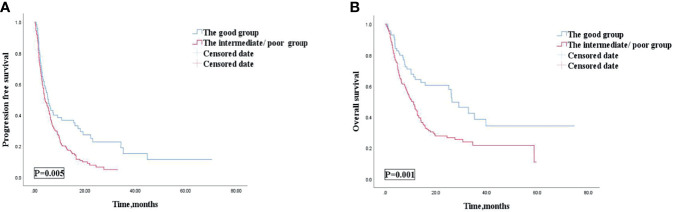
PFS **(A)** and OS **(B)** according to the good group and the intermediate/poor group of patients with AGC receiving ICIs cohort. PFS, progression free survival; OS, overall survival; AGC, advanced gastric cancer; ICIs, immune checkpoint inhibitors.

Among 238 AGC patients, 203 (85.3%) patients had tumor progression by the last follow-up date of July 1, 2021. The median PFS was 4.7 (95% CI: 3.686–5.714) months (**Table 4**). After we checked for hazard proportionality, Cox regression multivariable approach was performed (**Supplementary Figure 2A**). Univariate and multivariate analyses of factors associated with PFS were shown in **Table 5**. In univariate analysis, patients with a good dNLR/PLR score, with fewer organ metastases (<3), with a good PS (ECOG PS of 0–1), with no ascites, or with no pleural fluid showed improved PFS. Moreover, patients who did not reach PD before immunotherapy, who were treated with the 1st line ICIs, who were treated with more doses of ICIs (≥200 mg), or who were treated with ICIs combined with other therapies were also associated with improved PFS. Patients in the good dNLR/PLR group were closely related to longer PFS, compared to those in the poor dNLR/PLR group (5.5 months vs. 4.1 months; *p* = 0.005) (**Figure 3A** and **Table 4**). Multivariate analysis revealed that patients in the intermediate/poor dNLR/PLR group were independently correlated with an over 1.4 times greater risk of disease progression (HR = 1.499; 95% CI, 1.078–2.086; *p* = 0.016) than those in the good dNLR/PLR group. In addition, we also noticed that patients in the intermediate dNLR/PLR group were closely related to longer PFS, compared to those in the poor dNLR/PLR group (5.8 months vs. 3.8 months). In other words, the intermediate group was correlated with an over 1.3 times greater risk of disease progression (HR = 1.394; 95% CI, 1.009–1.926; *p* = 0.044) than the poor group (**Table 6**). Multivariate analysis revealed that patients in the intermediate/poor dNLR/PLR group were independently correlated with an over 1.4 times greater risk disease progression (HR = 1.499; 95% CI, 1.078–2.086; *p* = 0.016) than those in the good NLR/PLR group. Moreover, patients who had fewer organ metastases (<3) and treated with the 1st line ICIs were independently associated with improved PFS. Additionally, patients who had more organ metastases (≥3) were independently correlated with an over 1.5 times greater risk of disease progression (HR = 1.581; 95% CI, 1.108–2.256; *p* = 0.011) than those that had fewer organ metastases (<3). Moreover, patients treated with ICIs after 1st line were independently correlated with an over 2.3 times greater risk of disease progression (HR = 2.355; 95% CI, 1.645–3.370; *p* < 0.001) than those treated with the 1st line ICIs.

**Table 4:** Efficacy and prognosis based on the good and the intermediate/poor groups.

dNLR combined with PLR score classification		Response rate		OS (months)		PFS (months)	
		DCR (*n*, %)	OR (95% CI)	Median	HR (95% CI)	Median	HR (95% CI)
Overall	(*n* = 238)	126 (52.9)		12.5 (10.278–14.722)		4.7 (3.686–5.714)	
The good group	(*n* = 71)	40 (56.3)	1 [the reference]	26.3 (18.895–33.705)	1 [the reference]	5.5 (3.787–7.213)	1 [the reference]
The intermediate/poor group	(*n* = 167)	86 (51.5)	0.823 (0.471–1.439)	11.1 (8.823–13.377)	1.909 (1.299–2.805)	4.1 (2.961–5.239)	1.582 (1.147–2.181)
*p*-value		0.494		0.001		0.005	

PFS, progression-free survival; OS, overall survival; HR, hazard ratio; DCR, disease control rate; OR, odds ratio.

**Table 5:** Univariate and multivariate analyses of factors associated with PFS and OS.

Patient Characteristics	Univariate Analysis		Multivariate Analysis	
PFS	HR (95% CI)	*p*	HR (95% CI)	*p*
Lines of immunotherapy				
<2	1 [the reference]	<0.001	1 [the reference]	<0.00
≥2	2.402 (1.798–3.208)		2.387 (1.783–3.196)	1
Pleural fluid				
Absent	1 [the reference]	0.017	1 [the reference]	0.055
Present	2.062 (1.140–3.727)		1.792 (0.987–3.252)	
Ascites				
Absent	1 [the reference]	0.012	1 [the reference]	0.674
Present	1.511 (1.094–2.087)		1.081 (0.753–1.550)	
ECOG PS				
0–1	1 [the reference]	0.002	1 [the reference]	0.376
≥ 2	2.316 (1.363–3.936)		1.310 (0.721–2.379)	
Dosage of immunotherapy, median				
<200 mg	1 [the reference]	0.026	1 [the reference]	0.557
≥200 mg	1.375 (1.040–1.817)		1.098 (0.804–1.499)	
Response to line before immunotherapy				
Others	1 [the reference]	0.007	1 [the reference]	0.857
PD	1.518 (1.120–2.058)		0.968 (0.684–1.372)	
Number of metastatic sites				
<3	1 [the reference]	0.020	1 [the reference]	0.027
≥3	1.458 (1.061–2.003)		1.439 (1.042–1.987)	
ICIs combined with other therapies				
Yes	1 [the reference]	0.024	1 [the reference]	0.541
No	1.495 (1.050–2.000)		1.116 (0.785–1.584)	
dNLR combined with PLR score				
The good group	1 [the reference]	0.005	1 [the reference]	0.016
The intermediate/poor group	1.582 (1.147–2.181)		1.499 (1.078–2.086)	
**Patient Characteristics**	**Univariate Analysis**		**Multivariate Analysis**	
**OS**	**HR (95% CI)**	***p***	**HR (95% CI)**	***p***
ECOG PS				
0–1	1 [the reference]	<0.001	1 [the reference]	0.028
≥ 2	4.251 (2.471–7.312)		1.937 (1.075–3.489)	
Lines of immunotherapy				
<2	1 [the reference]	<0.001	1 [the reference]	<0.00
≥2	2.668(1.881–3.785)		2.355 (1.645–3.370)	1
Bone metastasis				
Absent	1 [the reference]	0.019	1 [the reference]	0.186
Present	1.782(1.100–2.887)		1.453 (0.835–2.527)	
Ascites				
Absent	1 [the reference]	0.031	1 [the reference]	0.785
Present	1.485 (1.037–2.125)		1.056 (0.715–1.560)	
Pleural fluid				
Absent	1 [the reference]	0.035	1 [the reference]	0.315
Present	2.002(1.050–3.815)		1.445 (0.705–2.961)	
Number of metastatic sites				
<3	1 [the reference]	0.002	1 [the reference]	0.011
≥3	1.765 (1.242–2.507)		1.581 (1.108–2.256)	
Response to line before immunotherapy				
Others	1 [the reference]	0.001	1 [the reference]	0.404
PD	1.896 (1.302–2.760)		1.204 (0.779–1.862)	
Dosage of immunotherapy, median (range)				
≥200 mg	1 [the reference]	<0.001	1 [the reference]	0.005
<200 mg	1.984 (1.440–2.734)		1.625 (1.156–2.286)	
ICIs combined with other therapies				
Yes	1 [the reference]	0.001	1 [the reference]	0.760
No	1.825 (1.280–2.603)		1.064 (0.713–1.588)	
dNLR combined with PLR score				
The good group	1 [the reference]	0.001	1 [the reference]	0.033
The intermediate/poor group	1.909 (1.299–2.805)		1.540 (1.036–2.288)	

PFS, progression-free survival; OS, overall survival; AGC, advanced gastric cancer; ICIs, immune checkpoint inhibitors; HR, hazard ratio; dNLR, derived neutrophil‐to‐lymphocyte, PLR, platelet–lymphocyte ratio; PD, progressive disease; ECOG PS, Eastern Cooperative Oncology Group performance status scores.

**Table 6:** Efficacy and prognosis based on the good, the intermediate, and the poor groups.

dNLR combined with PLR score classification		Response rate		OS (months)		PFS (months)	
		DCR (*n*, %)	OR (95% CI)	Median	HR (95% CI)	Median	HR (95% CI)
Overall	(*n* = 238)	126 (52.9)		12.5 (10.278–14.722)		4.7 (3.686–5.714)	
The good group	(*n* = 71)	40 (56.3)	1 [the reference]	26.3 (19.029–33.571)	1 [the reference]	5.5 (3.787–7.213)	1 [the reference]
The intermediate group	(*n* = 86)	43 (50.0)	1.290 (0.686–2.426)	12.1 (10.687–13.513)	1.540 (0.999–2.373)	5.8 (3.437–8.163)	1.355 (0.944–1.944)
The poor group	(*n* =81)	43 (53.1)	1.140 (0.601–2.164)	8.2 (4.966–11.434)	2.406 (1.579–3.665)	3.8 (3.017–4.583)	1.889 (1.319–2.704)
*P*-value		0.730		<0.001		0.002	
The intermediate group	(*n* = 86)	43 (50.0)	1 [the reference]	12.1 (10.687–13.513)	1 [the reference]	5.8 (3.437–8.163)	1 [the reference]
The poor group	(*n* =81)	43 (53.1)	0.884 (0.481–1.622)	8.2 (4.966–11.434)	1.562 (1.083–2.253)	3.8 (3.017–4.583)	1.394 (1.009–1.926)
*p*-value		0.690		0.017		0.044	
The good group	(*n* = 71)	40 (56.3)	1 [the reference]	26.3 (19.029–33.571)	1 [the reference]	5.5 (3.787–7.213)	1 [the reference]
The intermediate group	(*n* = 86)	43 (50.0)	1.290 (0.686–2.426)	12.1 (10.687–13.513)	1.540 (0.999–2.373)	5.8 (3.437–8.163)	1.355 (0.944–1.944)
*p*-value				0.050		0.099	
The good group	(*n* = 71)	40 (56.3)	1 [the reference]	26.3 (19.029–33.571)	1 [the reference]	5.5 (3.787–7.213)	1 [the reference]
The poor group	(*n* =81)	43 (53.1)	1.140 (0.601–2.164)	8.2 (4.966–11.434)	2.406 (1.579–3.665)	3.8 (3.017–4.583)	1.889 (1.319–2.704)
*p*-value				<0.001		0.001	

PFS, progression-free survival; OS, overall survival; HR, hazard ratio; DCR, disease control rate; OR, odds ratio.

### The Composite Biomarker of dNLR and PLR for OS of AGC Patients

Among 238 AGC patients, 150 (63%) patients died by the last follow-up date of July 1, 2021. The mOS was 12.5 (95% CI, 10.278–14.722) months (**Table 4**). After we checked for hazard proportionality, Cox regression multivariable approach was performed (**Supplementary Figure 2B**). Univariate and multivariate analyses of factors associated with OS are shown in **Table 5**. In univariate analysis, patients with a good dNLR/PLR score, with fewer organ metastases (<3), with a good PS (ECOG PS of 0–1), with no bone metastasis, with no ascites, or with no pleural fluid showed improved OS. Moreover, patients who did not reach PD before immunotherapy, and those who were treated with ICIs combined with other therapies, who were treated with the 1st line ICIs, who were treated with more doses of ICIs (≥200 mg), or who treated with ICIs combined with other therapies were also associated with improved OS. Patients in the good dNLR/PLR group were closely related to longer OS, compared to those in the intermediate/poor dNLR/PLR group (26.3 months vs. 11.1 months; *p* = 0.001) (**Figure 3B** and **Table 4**). In addition, we also noticed that patients in the intermediate dNLR/PLR group were closely related to longer OS, compared to those in the poor dNLR/PLR group (12.1 months vs. 8.2 months). In other words, the intermediate group was correlated with an over 1.56 times greater risk of death (HR = 1.562; 95% CI, 1.083–2.253; *p* = 0.017) than the poor group (**Table 6**). Multivariate analysis revealed that patients in the intermediate/poor dNLR/PLR group were independently correlated with an over 1.54 times greater risk of death (HR = 1.540; 95% CI, 1.036–2.288; *p* = 0.033) than those in the good dNLR/PLR group. Moreover, patients with fewer organ metastases (<3) or with a good PS (ECOG PS of 0–1) were independently associated with improved OS. Furthermore, patients who were treated with the 1st line ICIs or who were treated with more doses of ICIs (≥200 mg) were also independently associated with improved OS. Firstly, patients who had more organ metastases (≥3) were independently correlated with an over 1.5 times greater risk of death (HR = 1.581; 95% CI, 1.108–2.256; *p* = 0.011) than those who had fewer organ metastases (<3). Moreover, patients who had a good PS (ECOG PS of 0–1) were independently correlated with an over 1.9 times greater risk of death (HR = 1.937; 95% CI, 1.075–3.489; *p* = 0.028) than those had a poor PS (ECOG PS of ≥2). Furthermore, patients treated with ICIs after 1st line were independently correlated with an over 2.3 times greater risk of death (HR = 2.355; 95% CI, 1.645–3.370; *p* < 0.001) than those treated with the 1st line ICIs. Patients treated with less doses of ICIs (<200 mg) were independently correlated with an over 1.6 times greater risk of death (HR = 1.625; 95% CI, 1.156–2.286; *p* = 0.005) than those treated with more doses of ICIs (≥200 mg).

### Association of the Composite Biomarker of dNLR and PLR With Outcomes in Lines of Immunotherapy of 1 or a Large Number of Lines of Immunotherapy (≥2): Subgroup Analysis

**Figure 4:**
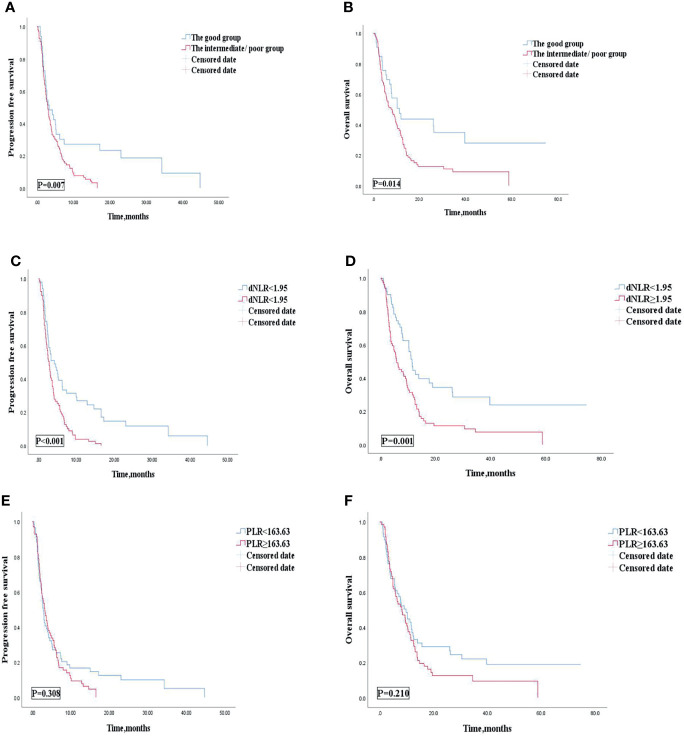
PFS **(A)** and OS **(B)** of the good group and the intermediate/poor group of the multi-line of patients with AGC, receiving ICIs cohort, PFS **(C)** and OS **(D)** of the dNLR of the multi-line of patients with AGC, receiving ICIs cohort, and PFS **(E)** and OS **(F)** of the PLR of the multi-line of patients with AGC, receiving ICIs cohort. PFS, progression-free survival; OS, overall survival; ICIs, immune checkpoint inhibitors; dNLR, derived neutrophil‐to‐lymphocyte; PLR, platelet–lymphocyte ratio.

Multivariate analysis revealed that patients treated with the 1st line ICIs were independently correlated with improved OS and PFS. Our study then conducted subgroup analysis based on different lines of immunotherapy. Univariate analyses of association of the dNLR/PLR group with outcomes in a large number of lines of immunotherapy (≥2) are shown in **Table 7**. For 130 patients treated with ICIs in subsequent lines, 97 (58.1%) patients were in the intermediate/poor dNLR/PLR group and 33 (46.5%) patients were in the good group. The median PFS and OS were 8.4 and 3 months, respectively. Patients of the intermediate/poor dNLR/PLR group had shorter PFS and OS than the good dNLR/PLR group (2.9 months vs. 3.3 months, 8.1 months vs. 11.2 months; *p* = 0.007, *p* = 0.014) (**Figures 4A, B**, and **Table 7**). Moreover, Kaplan–Meier analysis show that patients using ICIs in multilines with an elevated dNLR value (≥1.95) had shorter PFS and OS than those with a lower dNLR value (2.8 months vs. 4.2 months, 5.8 months vs. 11.6 months; *p* < 0.001, *p* = 0.001) (**Figures 4C, D**). However, no clear differences in PFS and OS were observed between the patients with an elevated PLR value (≥163.63) and those with a lower PLR value (<163.63) (2.8 months vs. 3.2 months, 8.2 months vs. 9.7 months; *p* = 0.308, *p* = 0.210) (**Figures 4E, F**).

**Table 7:** Univariate analyses of the good group and the intermediate/poor group associated with OS and PFS of AGC patients treated with ICIs in the multi-line.

dNLR combined with PLR score classification	Patients treated with ICIs in the multi-line			
	OS (months)		PFS (months)	
	Median	HR (95%CI)	Median	HR (95%CI)
The good	11.2	1 [Reference]	3.3	1 [Reference]
The intermediate/ poor group	8.1	1.811 (1.117-2.935)	2.9	1.844 (1.168-2.909)
P value		0.014		0.007

PFS, progression free survival; OS, overall survival; AGC, advanced gastric cancer; ICIs, immune checkpoint inhibitors; HR, hazard ratio; dNLR, derived neutrophil‐to‐lym‐ phocyte: PLR, platelet-lymphocyte ratio.

**Figure 5:**
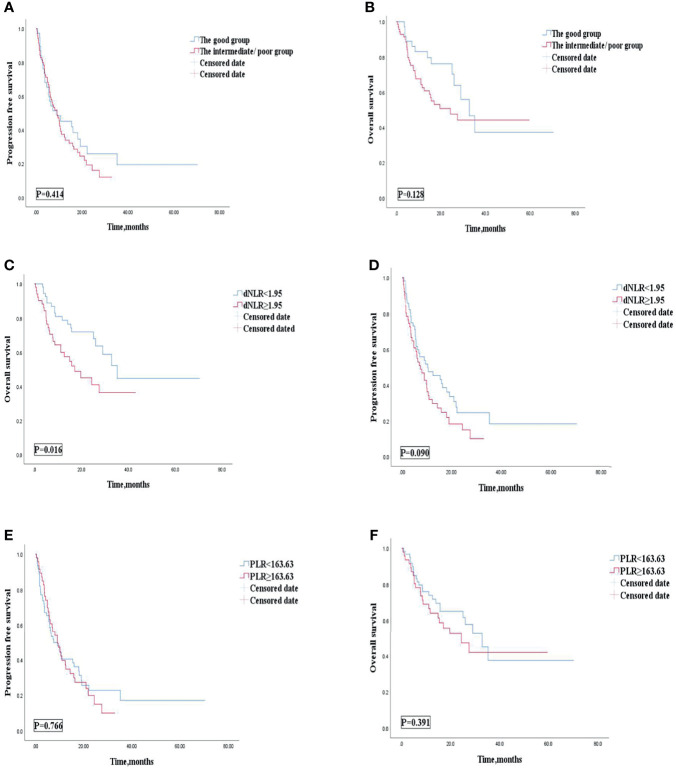
PFS **(A)** and OS **(B)** of the good group and the intermediate/poor group of the 1st line of patients with AGC, receiving ICIs cohort, OS **(C)** and PFS **(D)** of the dNLR of the 1st line of patients with AGC, receiving ICIs cohort, and OS **(E)** and PFS **(F)** of the PLR of the 1st line of patients with AGC, receiving ICIs cohort. PFS, progression-free survival; OS, overall survival; ICIs, immune checkpoint inhibitors; dNLR, derived neutrophil‐to‐lymphocyte; PLR, platelet–lymphocyte ratio.

Univariate analyses of association of the dNLR/PLR group with outcomes in lines of immunotherapy of 1 are shown in **Table 8**. For the 108 patients treated with ICIs in the 1st line, 70 (41.9%) patients were in the intermediate/poor dNLR/PLR group and 38 (53.5%) patients were in the good dNLR/PLR group. The median PFS and OS were 9.1 and 29 months, respectively. No clear differences in PFS and OS were observed between the intermediate/poor dNLR/PLR group and the good dNLR/PLR group (9.1 months vs. 9.1 months, 24.4 months vs. 32.8 months; *p* = 0.414, *p* = 0.128) (**Figures 5A, B**, and **Table 8**). Moreover, Kaplan–Meier analysis show that patients implementing ICIs in the 1st line with an elevated dNLR value (≥1.95) had shorter OS than those with a lower dNLR value (17.1 months vs. 35.2 months; *p* = 0.016) (**Figure 5C**). However, no clear difference of PFS was observed between the patients with an elevated dNLR value and with a lower dNLR value (7.6 months vs. 10.5 months; *p* = 0.090) (**Figure 5D**). Furthermore, there were no statistical differences in PFS and OS between the patients with an elevated PLR value (≥163.63) and with a lower PLR value (<163.63) (7.6 months vs. 9.1 months, 24.4 months vs. 32.8 months; *p* = 0.766, *p* = 0.391) (**Figures 5E, F**).

**Table 8:** Univariate analyses of the good group and the intermediate/poor group associated with OS and PFS of AGC patients treated with 1^st^ line ICIs.

dNLR combined with PLR score classification	Patients treated with the 1st line ICIs			
	OS (months)		PFS (months)	
	Median	HR (95%CI)	Median	HR (95%CI)
The good	32.8	1 [Reference]	9.1	1 [Reference]
The intermediate/ poor group	24.4	1.641 (0.861-3.127)	9.1	1.219 (0.756-1.963)
P value		0.128		0.414

PFS, progression free survival; OS, overall survival; AGC, advanced gastric cancer; ICIs, immune checkpoint inhibitors; HR, hazard ratio.

### Association of the Composite Biomarker of dNLR and PLR With Outcomes in ICIs Combined With Other Therapies or ICIs Monotherapy: Subgroup Analysis

**Figure 6:**
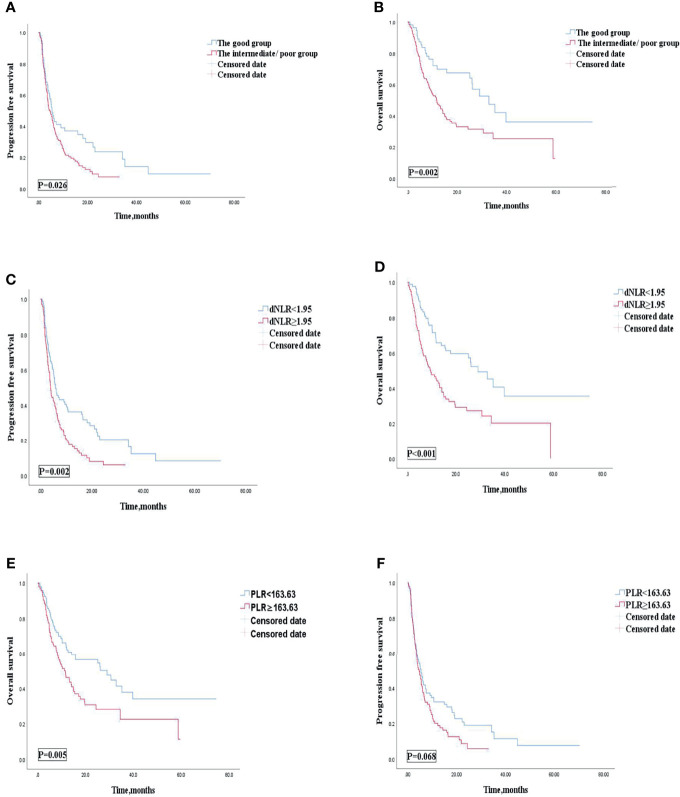
PFS **(A)** and OS **(B)** of the good group and the intermediate/poor group of patients with AGC, receiving ICIs combined with other therapies cohort, OS **(C)** and PFS **(D)** of the dNLR of patients with AGC, receiving ICIs combined with other therapies cohort, and OS **(E)** and PFS **(F)** of the PLR of patients with AGC, receiving ICIs combined with other therapies cohort. PFS, progression-free survival; OS, overall survival; ICIs, immune checkpoint inhibitors; dNLR, derived neutrophil‐to‐lymphocyte; PLR, platelet–lymphocyte ratio.

Univariate analysis revealed that patients treated with ICIs combined with other therapies were correlated with improved OS and PFS. Our study then conducted subgroup analysis based on ICIs combined with other therapies or ICI monotherapy. Univariate analyses of association of the dNLR/PLR group with outcomes in patients treated with ICIs combined with other therapies are shown in **Table 9**. For 186 patients in whom ICIs are combined with other therapies, 129 (69.4) patients were in the intermediate/poor dNLR/PLR group and 57 (30.6%) patients were in the good group. The median PFS and OS were 5.1 and 14.2 months, respectively. Patients of the intermediate/poor dNLR/PLR group had shorter PFS and OS than the good dNLR/PLR group (4.7 months vs. 5.5 months, 24.4 months vs. 32.8 months; *p* = 0.026, *p* = 0.002) (**Table 9**). Patients in the intermediate/poor dNLR/PLR group were correlated with an over 2 times greater risk of death (HR = 2.063; 95% CI, 1.305–3.260; *p* = 0.002) and with an over 1.5 times greater risk disease progression (HR = 1.507; 95% CI, 1.046–2.170; *p* = 0.028) than those in the good NLR/PLR group (**Figures 6A, B**, and **Table 9**). Moreover, Kaplan–Meier analysis shows that patients using ICIs combined with other therapies with an elevated dNLR value (≥1.95) had shorter PFS and OS than those with a lower dNLR value (3.9 months vs. 5.8 months, 9.5 months vs. 29.0 months; *p* = 0.002, *p* < 0.001) (**Figures 6C, D**). Furthermore, patients with an elevated PLR value (≥163.63) had shorter OS than those with a lower PLR value (<163.63) (11.6 months vs. 29.0 months; *p* = 0.005) (**Figure 6E**). However, no clear difference in PFS was observed between the patients with an elevated PLR value and with a lower PLR value (4.7 months vs. 5.5 months; *p* = 0.068) (**Figure 6F**).

**Table 9:** Univariate analyses of the good group and the intermediate/poor group associated with OS and PFS of AGC patients treated with ICIs combined with other therapies.

dNLR combined with PLR score classification	Patients treated with ICIs combined with other therapies.				
	Overall *n* = 186	OS (months)		PFS (months)	
		Median	HR (95% CI)	Median	HR (95% CI)
The good	57 (30.6)	32.8	1 [Reference]	5.5	1 [Reference]
The intermediate/poor group	129 (69.4)	11.8	2.063 (1.305–3.260)	4.7	1.507 (1.046–2.170)
*p*-value		0.002		0.028	

PFS, progression-free survival; OS, overall survival; AGC, advanced gastric cancer; ICIs, immune checkpoint inhibitors; HR, hazard ratio; dNLR, derived neutrophil‐to‐lymphocyte, PLR, platelet–lymphocyte ratio.

**Figure 7:**
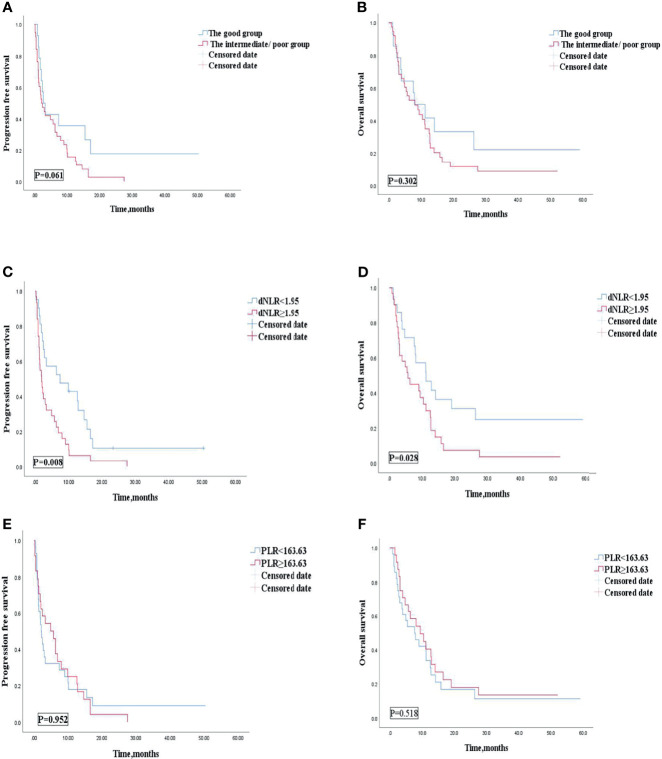
PFS **(A)** and OS **(B)** of the good group and the intermediate/poor group of patients with AGC, receiving ICIs monotherapy cohort, OS **(C)** and PFS **(D)** of the dNLR of patients with AGC, receiving ICIs monotherapy cohort, and OS **(E)** and PFS **(F)** of the PLR of patients with AGC, receiving ICIs monotherapy cohort. PFS, progression-free survival; OS, overall survival; ICIs, immune checkpoint inhibitors; dNLR, derived neutrophil‐to‐lymphocyte; PLR, platelet–lymphocyte ratio.

Univariate analyses of association of the dNLR/PLR group with outcomes in patients treated with ICIs monotherapy are shown in **Table 10**. For the 52 patients treated with ICI monotherapy, 38 (73.1%) patients were in the intermediate/poor dNLR/PLR group and 14 (26.9%) patients were in the good dNLR/PLR group. The median PFS and OS were 2.4 and 8.1 months, respectively. No clear differences in PFS and OS were observed between the intermediate/poor dNLR/PLR group and the good dNLR/PLR group (2.2 months vs. 2.7 months, 8.1 months vs. 7.9 months; *p* = 0.061 *p* = 0.302) (**Figures 7A, B**, and **Table 10**). Moreover, Kaplan–Meier analysis shows that patients in whom ICIs were implemented in the multiline with an elevated dNLR value (≥1.95) had shorter PFS and OS than those with a lower dNLR value (1.9 months vs. 7.4 months, 5.7 months vs. 11.2 months; *p* = 0.008, *p* = 0.028) (**Figures 7C, D**). However, no clear differences in PFS and OS were observed between the patients with an elevated PLR value (≥163.63) and those with a lower PLR value (<163.63) (2.1 months vs. 4.9 months, 7.6 months vs. 9.4 months; *p* = 0.952, *p* = 0.518) (**Figures 7E, F**).

**Table 10:** Univariate analyses of the good group and the intermediate/poor group associated with OS and PFS of AGC patients treated with ICIs monotherapy.

dNLR combined with PLR score classification	Patients treated with ICIs combined without chemotherapy				
	Overall *n* = 52	OS (months)		PFS (months)	
		Median	HR (95% CI)	Median	HR (95% CI)
The good	14 (26.9)	7.9	1 [Reference]	2.7	1 [Reference]
The intermediate/poor group	38 (73.1)	8.1	1.448 (0.711–2.948)	2.2	1.887 (0.953–3.738)
*p*-value		0.307		0.069	

PFS, progression-free survival; OS, overall survival; AGC, advanced gastric cancer; ICIs, immune checkpoint inhibitors; HR, hazard ratio; dNLR, derived neutrophil‐to‐lymphocyte; PLR, platelet–lymphocyte ratio.

## Discussion

The usage of immunotherapy in the field of GC treatment has increased annually worldwide (7, 21). Based on the current clinical trial results, the positive effect of immune checkpoint inhibitors is very apparent (22). Comparatively, immunotherapy drugs are expensive and prone to drug resistance and even super-progress (23, 24). Therefore, finding an effective predictive marker is an urgent matter to be solved. These indicators can predict immune curative effect, so as to achieve precise treatment. However, the current evaluation of biomarkers for immunotherapy is relatively limited (15). The highly heterogeneous characteristics of GC may limit the accuracy of a single biomarker for screening populations benefiting from immunotherapy (25). In contrast, the combination of multiple indicators can provide more targeted information for the detection of potential immune benefit subgroups. Peripheral inflammatory blood indexes such as NLR, dNLR, and PLR are independent prognostic biomarkers for patients receiving immunotherapy (13, 14, 20). A prognostic correlation analysis of patients with advanced non-small cell lung cancer treated with peripheral blood biomarkers and anti-PD-1 antibody treatment by Soyano et al. showed that patients with an elevated PLR value were correlated independently with poor prognosis (26). When the fluctuations of PLR are interpreted along with other complementary hematologic indices, its value as an inflammatory marker will increase. One typical example of the complementary hematologic index is NLR, which provides additional information about neutrophilic inflammation and infectious complications (27). Consequently, Dharmapuri et al. established a statistical model by NLR/PLR groups and found that there were significant differences in survival between the high-NLR/low-PLR group and the low-NLR/low-PLR group in advanced hepatocellular carcinoma patients treated with ICIs (16). The efficiency of dNLR as useful biomarkers, predicting ICI response, has been proved by Lim et al. (20). Our research also found that patients with an elevated dNLR value (≥ the best cutoff value) were associated with shorter OS and PFS. Patients with an elevated dNLR value were associated with an over 1.8 times greater risk of disease progression (HR = 1.807; 95% CI, 1.356–2.407; *p* < 0.001) and an over 2.1 times greater risk of death (HR = 2.161; 95% CI, 1.542–3.028; *p* < 0.001) than those with a lower dNLR value. However, patients with high levels of PLR (≥ the median value) were only associated with shorter OS, but not PFS. Patients with an elevated PLR value were associated with an over 1.4 times greater risk of death than those with a lower PLR value (<163.63) (HR = 1.416; 95% CI, 1.026–1.956; *p* = 0.033). On the other hand, Baicun Hou et al. noticed that the Lung immune prognostic index (LIPI), consisting of lactate dehydrogenase (LDH) levels and dNLR, was correlated with the outcomes of AGC patients receiving immunotherapy (28). As such, we combined dNLR and PLR to stratify risk factors. The high levels of dNLR (≥1.95) and PLR (≥163.63) were considered to be risk factors. Based on these two risk factors, patients were categorized into 3 groups: the risk factor number for the “good” group was 0, that for the “intermediate” group was 1, and that for the “poor” group was 2. Due to the similar efficacy and survival outcomes of patients in intermediate and good groups, the subjects were divided into two groups: dNLR/PLR-good and dNLR/PLR-intermediate/poor. We then began to evaluate the differences in prognosis and survival of AGC patients after immunotherapy between the good and the intermediate/poor groups. The cutoff value of dNLR was obtained by the ROC curves to predict the disease progression rate at the 8th month and the cutoff value of PLR was estimated by the median value. The cutoff values of dNLR and PLR were 1.95 and 163.63, respectively. Dharmapuri et al. found that the high-NLR/low-PLR group has shorter OS and PFS than the low-NLR/low-PLR group. We also found that the good dNLR/PLR group was independently associated with better prognosis. The intermediate/poor dNLR/PLR group was independently correlated with an over 1.4 times greater risk of disease progression (4.1 months vs. 5.5 months; *p* = 0.016) and an over 1.54 times greater risk of death (11.1 months vs. 26.3 months; *p* = 0.033) than the good dNLR/PLR group. However, no clear differences in the disease control rate (DCR) and overall response rate (ORR) were observed between the intermediate/poor dNLR/PLR group and the good dNLR/PLR group (51.5% vs. 56.3%, 26.3% vs. 29.6%; *p* = 0.494, *p* = 0.609). Baicun Hou et al. noticed that patients with a good PS (ECOG PS of 0–1) were also independently associated with PFS and OS for AGC patients treated with ICIs (28). However, in our study, patients who had a good PS (ECOG PS of 0–1) were independently associated with improved OS, but without improved PFS. Baicun Hou et al. noticed that patients treated with combination of immunotherapy and other therapies were associated with longer OS, with HRs of 0.58 (95% CI, 0.37–0.93; *p* = 0.024), and PFS, with HRs of 0.49 (95% CI, 0.30–0.81; *p* = 0.005) (28). However, in our study, there were no statistical differences for OS and PFS between patients treated with ICIs, ICI plus chemotherapy, ICI plus antiangiogenic and chemotherapy group, and ICI plus antiangiogenic or target agents. We found that patients treated with the 1st line ICIs were independently associated with improved PFS and OS. However, no clear differences in OS and PFS were observed between patients treated with the 1st line ICIs and those treated with ICIs after the 1st line in the study of Baicun Hou et al. (28). We also found that patients who had fewer organ metastases (<3) were independently associated with improved PFS and OS. However, Baicun Hou et al. found that patients who had fewer organ metastases (< 2) were not independently associated with improved PFS and OS than those who had more organ metastases (≥2) (28). In addition, our study firstly found that patients treated with more doses of ICIs (≥200 mg) were independently associated with improved OS, but without improved PFS. However, the mechanism of the correlation between this peripheral blood inflammatory complex index and the tumor prognosis is relatively complicated, and it still needs to be further explored through basic experiments and clinical trials. Some studies suggested that this may be related to the tumor-immune microenvironment of patients (29, 30). In addition to direct immune killing effects on tumor cells, these biomarkers are also related to tumor immunostimulatory signals and the activation of effector cells. Neutrophils are derived from bone marrow hematopoietic stem cells and have chemotaxis, phagocytosis, and bactericidal effect (31). Moreover, not only can it enhance the growth of tumor cells under the effect of tumor, its microenvironment reproduction and invasion can also promote angiogenesis and mediate tumor immunosuppression (32).

Lymphocyte is an important component for the body’s immune response function (33). Elevated neutrophils can inhibit the immune attack ability of lymphocytes (34). Consequently, NLR, defined as the ratio of neutrophils to lymphocytes, can comprehensively reflect the immune status and inflammation of the tumor patients (35). dNLR is defined as the ratio between the neutrophil and white blood cell minus neutrophil. The dNLR can reflect changes in the body’s immune system, so it is more meaningful than NLR (36). Platelets are produced by mature megakaryocytes in bone marrow hematopoietic tissue (37). It can release inflammatory factors such as thrombospondin and vascular endothelial growth factor, and participates in tumor cell adhesion, extravasation, invasion, immune escape, and tumor angiogenesis (38). Moreover, tumors grow and evolve through constant crosstalk with the surrounding microenvironment, and emerging evidence indicates that angiogenesis and immunosuppression frequently occur simultaneously in response to this crosstalk (39). Accordingly, strategies combining anti-angiogenic therapy and immunotherapy seem to have the potential to tip the balance of the tumor microenvironment and improve treatment response (39). Therefore, based on the value of PLR, we may be able to roughly assess whether patients can benefit from the therapy of ICI combined with antiangiogenic agents. dNLR and PLR are composite indicators of lymphocyte, neutrophil, and platelet, so they can reflect the balance of the body’s tumor inflammatory response to a certain extent. Therefore, the higher dNLR and PLR tumor patients get, the worse their prognosis will be.

As a retrospective data collection, this study might have some reporting errors. Due to these, positive results could be exaggerated, and some false positives could appear on research results. However, these errors were inevitable in research design. Moreover, this study had some limitations, including a relatively small sample size with a mixed population of GC of cardia, GC of body/fundus, and GC of pylorus, as well as a lack of comparison of the two groups among the three cancers.

## Conclusion

This retrospective cohort study has demonstrated that a composite biomarker of dNLR and PLR is independently correlated with the survival of AGC patients implementing immunotherapy. It may be difficult for patients with the intermediate/poor dNLR/PLR group to benefit from immunotherapy. However, the possibility of using the complex index as an effective and economic prognostic biomarker to select patients who are best suited to receiving ICIs needs further investigation in a larger prospective study.

## Data Availability Statement

The raw data supporting the conclusions of this article will be made available by the authors, without undue reservation.

## Ethics Statement

This study was approved by the Ethics Committee of Chinese PLA General Hospital and was conducted according to the principles of the Declaration of Helsinki.

## Author Contributions

YP was in charge of writing and analysis. GD and ZW provided the guide and idea. HS, GCD, SC, NZ, and QZ contributed to analysis. All authors contributed to the article and approved the submitted version.

## Conflict of Interest

The authors declare that the research was conducted in the absence of any commercial or financial relationships that could be construed as a potential conflict of interest.

## Publisher’s Note

All claims expressed in this article are solely those of the authors and do not necessarily represent those of their affiliated organizations, or those of the publisher, the editors and the reviewers. Any product that may be evaluated in this article, or claim that may be made by its manufacturer, is not guaranteed or endorsed by the publisher.

## Supplementary Material

Supplementary Table 1 Relationship between the good, the intermediate, and the poor groups and response to ICIs treatment. :response rate.

Supplementary Figure 1 PFS **(A)** and OS **(B)** according to the good,the intermediate, and the poor groups of patients with AGC receiving ICIs cohort. PFS, progression free survival; OS,overall survival; AGC,advanced gastric cancer; ICIs, immune checkpoint inhibitors.

Supplementary Figure 2 Cox proportional hazard of PFS **(A)** and OS **(B)**. PFS, progression free survival; OS, overall survival; AGC, advanced gastric cancer; ICIs, immune checkpoint inhibitors.

## Abbreviations

GC, Gastric cancer, AGC, advanced gastric cancer; ICIs, immune checkpoint inhibitors; OS, overall survival; PD-L1, programmed death ligand-1; MSI, microsatellite steady-state; NLR, neutrophil–lymphocyte ratio; dNLR, derived neutrophil‐to‐lymphocyte; PLR, platelet–lymphocyte ratio; Hb, hemoglobin; PFS, progression-free survival; ROC, receiver operating characteristic; ECOG PS, Eastern Cooperative Oncology Group performance status scores; ANC, absolute neutrophil count; ALC, absolute lymphocyte count; PLT, platelet count; DCR, disease control rate; CR, complete response; SD, stable disease; PR, partial response; ORR, overall response rate; PD, progressive disease; LDH, lactate dehydrogenase.
