# Supplementary material for: A Composite Biomarker of Derived Neutrophil–Lymphocyte Ratio and Platelet–Lymphocyte Ratio Correlates With Outcomes in Advanced Gastric Cancer Patients Treated With Anti-PD-1 Antibodies
Source: Front Oncol. 2022 Feb 18;11:798415. doi: 10.3389/fonc.2021.798415 (PMC8895371; doi:10.3389/fonc.2021.798415)
Supplement: Supplementary Table 1 — Relationship between the good, the intermediate, and the poor groups and response to ICIs treatment. :response rate. [file DataSheet_2.docx]

Supplementary Table1 Relationship between the good, the intermediate, and the poor group and response to anti-PD-1 treatment.

| Best Overall Respons | Number of Patients (%) | | | | P value |
| --- | --- | --- | --- | --- | --- |
|  | Overall  n =238 | the good group  n =71 | the intermediate group  n = 86 | the poor group  n = 81 |  |
| CR | 4(1.7) | 2(2.8) | 2(2.3) | 0(0) | 0.183 |
| PR | 62(26.1) | 20(28.2) | 24(27.9) | 18(22.2) | 0.626 |
| SD | 60(25.2) | 18(25.4) | 17(19.8) | 25(30.9) | 0.256 |
| PD | 112(47.1) | 31(43.7) | 43(50.0) | 38(46.9) | 0.730 |
| ORR | 65(27.3) | 21(29.6) | 26(30.2) | 18(22.2) | 0.447 |

| Supplementary Figure1: PFS (A) and OS (B) according to the good group, the intermediate group, and the poor group of patients with AGC receiving ICIs cohort. | | | |
| --- | --- | --- | --- |
| A |  | B |  |
|  | 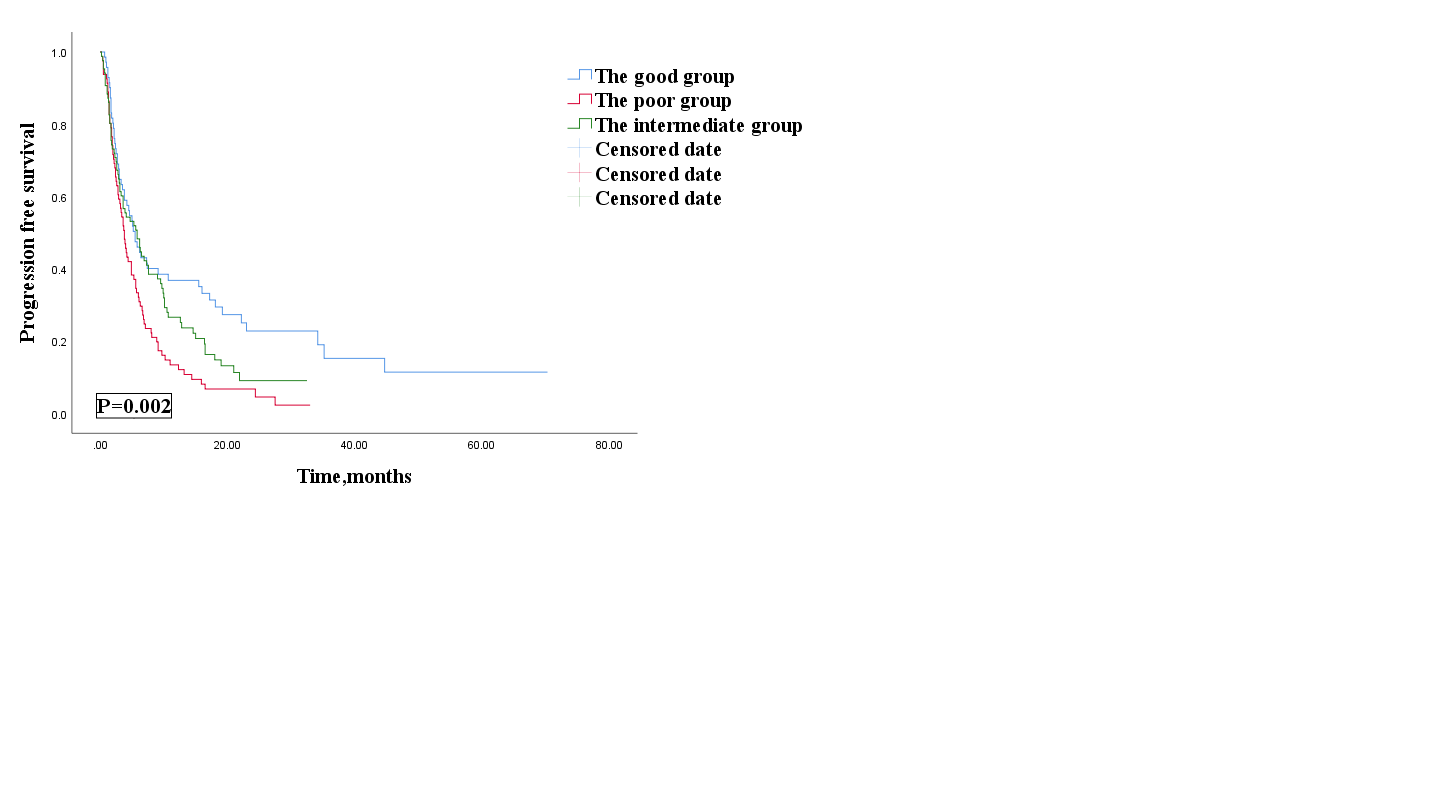 |  | 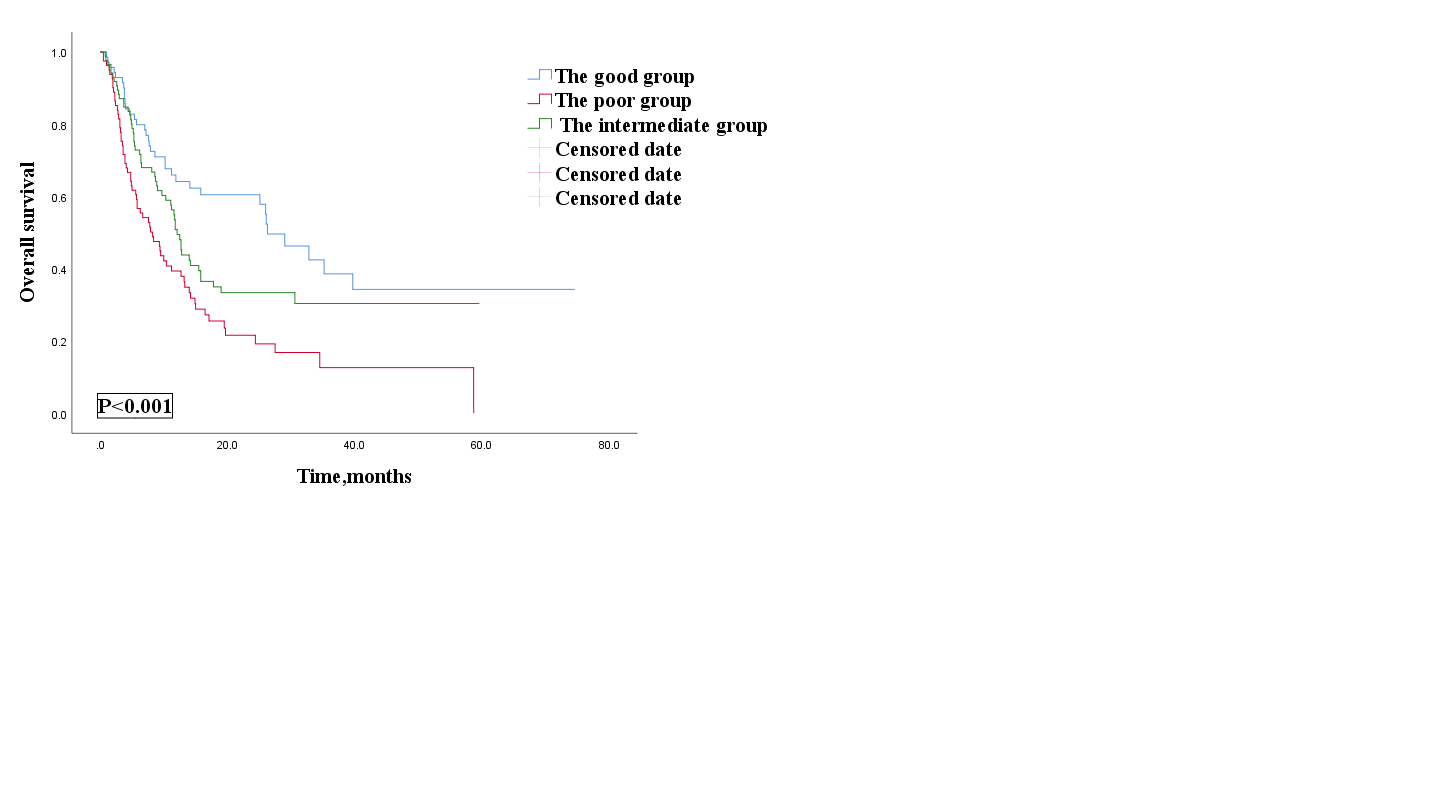 |
| Abbreviations: PFS: progression free survival; OS:overall survival; AGC:advanced gastric cancer; ICIs: immune checkpoint inhibitors;. | | | |

| Supplementary Figure 2: PFS (A) and OS (B) according to the good group, the intermediate group, and the poor group of patients with AGC receiving ICIs cohort. | |
| --- | --- |
|  | B |
| 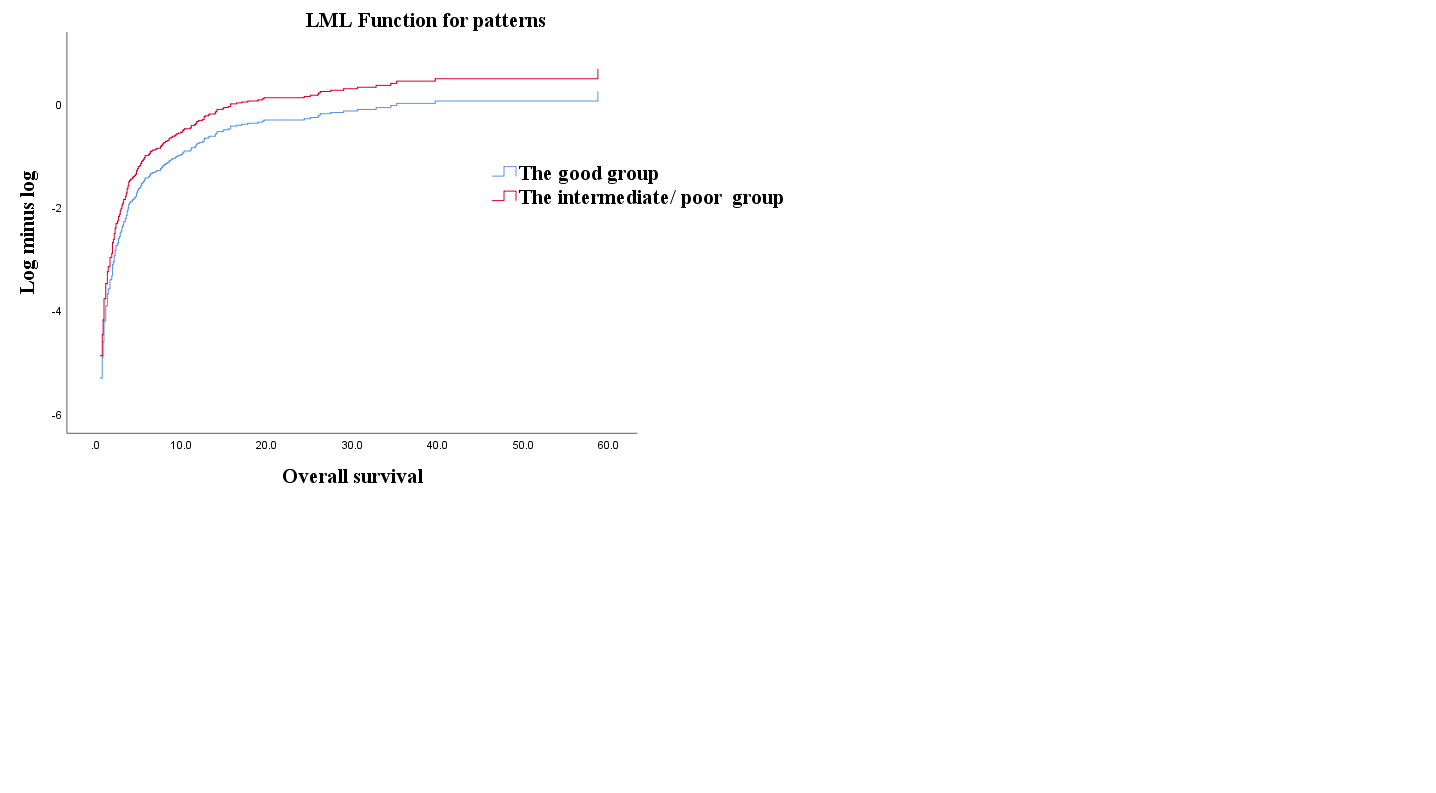 | 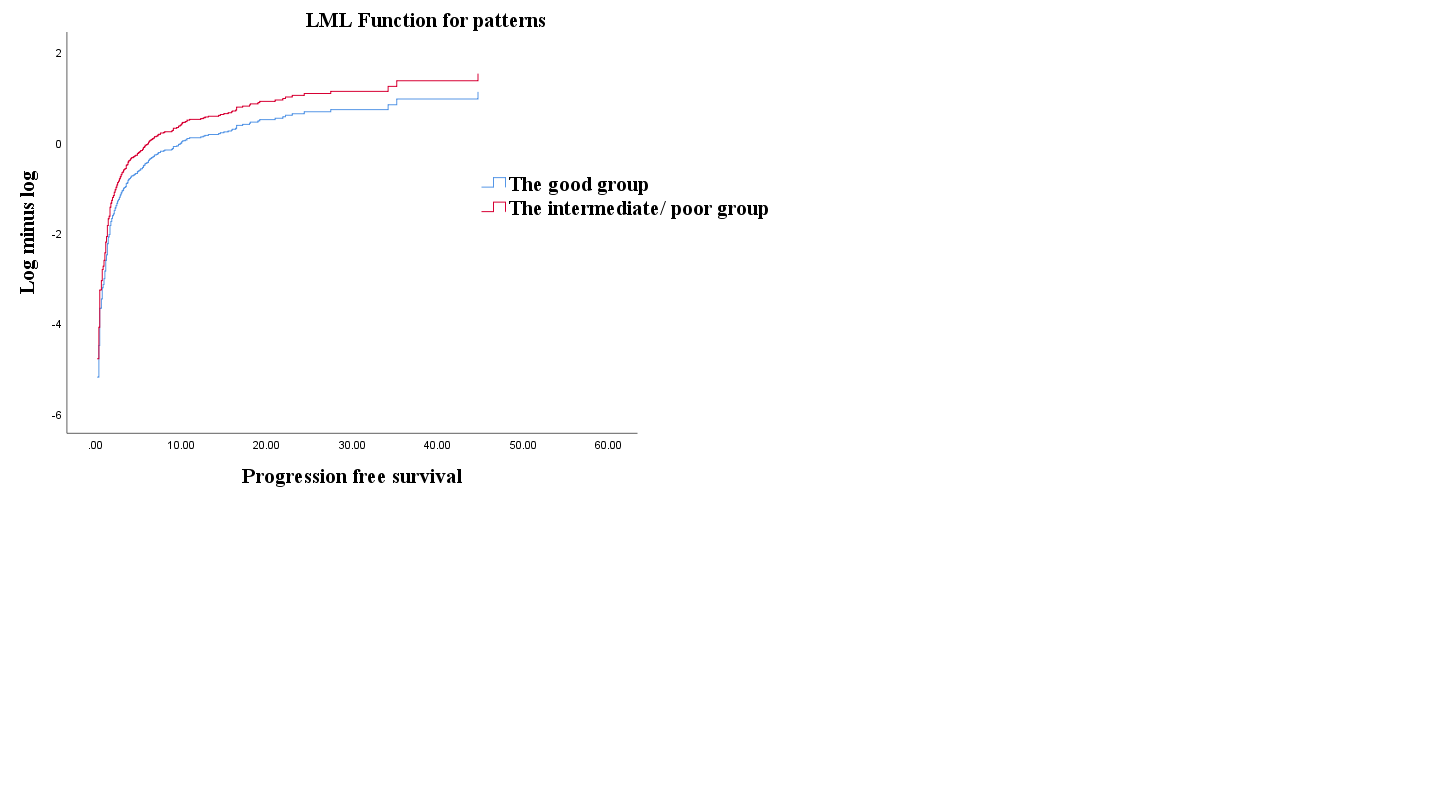 |
| Abbreviations: PFS: progression free survival; OS:overall survival; AGC:advanced gastric cancer; ICIs: immune checkpoint inhibitors;. | |
