# Supplementary material for: A Composite Biomarker of Derived Neutrophil–Lymphocyte Ratio and Platelet–Lymphocyte Ratio Correlates With Outcomes in Advanced Gastric Cancer Patients Treated With Anti-PD-1 Antibodies
Source: Front Oncol. 2022 Feb 18;11:798415. doi: 10.3389/fonc.2021.798415 (PMC8895371; doi:10.3389/fonc.2021.798415)
Supplement: Supplementary file 2 [file DataSheet_1.pdf]

Logrank Tests

Numeric Results for the Logrank Test in Terms of Sample Size
Alternative Hypothesis: Two-Sided

Power	N1	N2	N	Haz Ratio (HR)	Ctrl Med Surv Time (M1)	Trt Med Surv Time (M2)	Acc-rual Pat'n	Acc-rual Time/ Total Time	Ctrl Loss	Trt Loss	Ctrl to Trt	Trt to Ctrl	Alpha	Beta
0.8015	109	110	219	0.6471	11.00	17.00	Equal	77 / 79	0.0000	0.0000	0.0000	0.0000	0.0500	0.1985

References

Lakatos, Edward. 1988. 'Sample Sizes Based on the Log-Rank Statistic in Complex Clinical Trials', Biometrics, Volume 44, March, pages 229-241.
Lakatos, Edward. 2002. 'Designing Complex Group Sequential Survival Trials', Statistics in Medicine, Volume 21, pages 1969-1989.

Report Definitions
Power is the probability of rejecting a false null hypothesis. Power should be close to one.
N1|N2|N are the sample sizes of the control group, treatment group, and both groups, respectively.
Hazard Ratio (HR) is controls group's median survival time divided by the treatment group's median survival time.
Median Survival Time is the time until half the subjects fail.
Accrual Time is the number of time periods (years or months) during which accrual takes place.
Total Time is the total number of time periods in the study. Follow-up time = (Total Time) - (Accrual Time).
Ctrl Loss is the proportion of the control group that is lost (drop out) during a single time period (year or month).
Trt Loss is the proportion of the treatment group that is lost (drop out) during a single time period (year or month).
Ctrl to Trt (drop in) is the proportion of the control group that switch to a group with a hazard rate equal to the treatment group.
Trt to Ctrl (noncompliance) is the proportion of the treatment group that switch to a group with a hazard rate equal to the control group.
Alpha is the probability of rejecting a true null hypothesis. It should be small.
Beta is the probability of accepting a false null hypothesis. It should be small.

Numeric Results for the Logrank Test in Terms of Events
Alternative Hypothesis: Two-Sided

Power	Ctrl Evts (E1)	Trt Evts (E2)	Total Evts (E)	Haz Ratio (HR)	Ctrl Med Surv Time (T1)	Trt Med Surv Time (T2)	Acc-rual Pat'n	Acc-rual Time/ Total Time	Ctrl Loss	Trt Loss	Ctrl to Trt	Trt to Ctrl	Alpha	Beta
0.8015	89.8	78.7	168.5	0.6471	11.00	17.00	Equal	77 / 79	0.0000	0.0000	0.0000	0.0000	0.0500	0.1985

Summary Statements
A two-sided logrank test with an overall sample size of 219 subjects (109 in the control group and 110 in the treatment group) achieves 80.2% power at a 0.050 significance level to detect a hazard ratio of 0.6471 when the control group median survival time is 11.00. The study lasts for 79 time periods of which subject accrual (entry) occurs in the first 77 time periods. The accrual pattern across time periods is uniform (all periods equal). No subjects drop out of the control group. No subjects drop out of the treatment group. The proportion switching from the control group to another group with a median survival time equal to that of the treatment group is 0.0000. The proportion switching from the treatment group to another group with a median survival time equal to that of the control group is 0.0000.

Logrank Tests

Procedure Input Settings

Autosaved Template File
C:\Users\HUAWEI\Documents\PASS 15\Procedure Templates\Autosave\Logrank Tests - Autosaved 2021_11_27-20_4_17.t396

Design Tab

Solve For:	Sample Size
Alternative Hypothesis:	Two-Sided
Power:	0.80
Alpha:	0.05
Group Allocation:	Equal (N1 = N2)
Input Type:	Median Survival Time
T1 (Median Survival Time - Control):	11
Treatment Group Parameter:	T2 (Med. Survival Time - Treatment)
T2 (Median Survival Time - Treatment):	17
Accrual Time (Integers Only):	77
Accrual Pattern:	Uniform or Equal
Total Time (Integers Only):	79
Controls Lost:	0
Treatments Lost:	0
Controls Switch to Treatments:	0.0
Treatments Switch to Controls:	0.0

Options Tab

Number of Intervals within a Time Period:	2000
